# Central nervous system infection in a pediatric population in West Java

**DOI:** 10.1371/journal.pntd.0011769

**Published:** 2023-11-27

**Authors:** Dewi H. Alisjahbana, Syndi Nurmawati, Mia Milanti, Hofiya Djauhari, Jeremy P. Ledermann, Ungke Antonjaya, Yora Permata Dewi, Edison Johar, Ageng Wiyatno, Ida Yus Sriyani, Bachti Alisjahbana, Dodi Safari, Khin Saw Aye Myint, Ann M. Powers, Dzulfikar DL Hakim

**Affiliations:** 1 Department of Child Health, Hasan Sadikin Hospital, Faculty of Medicine, Universitas Padjadjaran, Bandung, Indonesia; 2 Research Center for Care and Control of Infectious Disease, Universitas Padjadjaran, Bandung, Indonesia; 3 Centers for Disease Control and Prevention, Fort Collins, Colorado, United States of America; 4 Oxford University Clinical Research Unit Indonesia, Faculty of Medicine Universitas Indonesia, Jakarta, Indonesia; 5 Emerging Virus Research Unit, Eijkman Institute for Molecular Biology, Jakarta, Indonesia; 6 Department of Internal Medicine, Hasan Sadikin Hospital, Faculty of Medicine, Universitas Padjadjaran, Bandung, Indonesia; 7 Eijkman Research Center for Molecular Biology, National Research and Innovation Agency, Jakarta, Indonesia; The University of Sydney School of Veterinary Science, AUSTRALIA

## Abstract

Central nervous system (CNS) viral infections are critical causes of morbidity and mortality in children; however, comprehensive data on etiology is lacking in developing countries such as Indonesia. To study the etiology of CNS infections in a pediatric population, 50 children admitted to two hospitals in Bandung, West Java, during 2017–2018 were enrolled in a CNS infection study. Cerebrospinal fluid and serum specimens were tested using molecular, serological, and virus isolation platforms for a number of viral and bacteriological agents. Causal pathogens were identified in 10 out of 50 (20%) and included cytomegalovirus (n = 4), *Streptococcus pneumoniae* (n = 2), tuberculosis (n = 2), *Salmonella* serotype Typhi (n = 1) and dengue virus (n = 1). Our study highlights the importance of using a wide range of molecular and serological detection methods to identify CNS pathogens, as well as the challenges of establishing the etiology of CNS infections in pediatric populations of countries with limited laboratory capacity.

## Author summary

Pediatric CNS infection is a high burden for public health in many developing countries including Indonesia where diagnostic capacity is limited. In this study we identify the clinical characteristics and etiology of CNS infection in 50 pediatric population from two hospitals in Indonesia. The data identified cytomegalovirus, dengue, *Salmonella* Typhi, *Streptococcus pneumoniae* and *Mycobacterium tuberculosis* as the etiology of infection while 80% of cases remain unidentified. The results are expected to raise awareness for policy makers to improve management and laboratory diagnosis of CNS infection in children. Further studies are required to identify contributing factors to the burden of CNS infection.

## Introduction

Central nervous system (CNS) infections are life-threatening and life-changing conditions in the pediatric population and are the most frequent cause of hospitalizations in children in many developing countries. However, there is a lack of data on the etiologies due to limited diagnostic capacity and the challenges associated with performing an investigative lumbar puncture. In spite of the difficulties, a number of pathogens have been documented to cause neurologic disease in developing countries. *Mycobacterium tuberculosis* (MTB) is the most common cause in adult and children in Indonesia [1,2]. Viruses, regarded as important etiological agents of encephalitis worldwide, vary with geographical location with vector borne viruses being most dominant in tropical developing countries [3]. Other viruses, including most human herpesviruses along with cytomegalovirus (CMV) are also known to be neurotropic [4]. *Haemophilus influenzae* type b (Hib) and *Streptococcus pneumoniae* (*S*. *pneumoniae*) are common causes of bacterial meningitis associated with high fatalities if not promptly treated [5]. With the aim to identify the main pathogens of acute meningoencephalitis (AME) in Indonesian children, we conducted a prospective hospital-based study over a one-year period using a number of diagnostic platforms to identify the potential range of viral and bacterial pathogens associated with CNS disease.

## Methods

### Ethics statement

The study was approved by the Institutional Ethics Committee on Medical Research Involving Human Subjects Dr. Hasan Sadikin Central General Hospital Bandung (LB.04.01/A05/EC/255/VIII/2017), and written informed consent was obtained from parents of the subjects.

### Study area and population

The study was conducted at two hospitals in Bandung, West Java, Indonesia. Bandung consists of urban and semi-urban areas, covering 167 square kilometers. Population size during the study was 2.4 million people with density of 14,608 people per square kilometer. The pediatric population (less than 19 years old) was approximately 759,000 or 31% of the total population. The city is located at 6° 54′ 43.2″S, 107° 36′ 34.92″E in a highland area 700 meters above sea level; the average temperature is 25.9°C and annual rainfall 200.4 mm. The city has resources to clean water with a relatively good sanitation and hygiene system compared to other cities in Indonesia [6].

### Study subjects

Patients between 6 months to 17 years admitted to the pediatric wards of Hasan Sadikin Hospital, a provincial top referral hospital and Ujung Berung Hospital, a lower referral level hospital in West Java between October 2017 and November 2018 with suspected CNS infection were enrolled based on the clinical judgment of admitting physicians to meet the study case definition as follows: febrile episode reported within the preceding month with at least one of the following signs: confusion; altered consciousness; seizures; or focal neurological deficit. Cerebrospinal fluid (CSF) and blood samples were obtained on admission, and a second serum sample was obtained on discharge. Throat and rectal swabs were not collected. Detailed demographic and clinical data, as well as blood and CSF biochemical and bacteriological data were collected. Short-term follow up for neurological sequelae and the final outcome was conducted at approximately 3 months after enrollment. Clinical outcomes were defined as death, full recovery, or presence of neurological sequelae on discharge and at a follow up. The 3-month hospital or home visit follow-up for neurological sequelae consisted of history taking and physical examination for focal neurological deficits to inquire for presence of any neurological symptoms.

### Laboratory testing algorithm

Every patient underwent standard of care tests or primary screening at the hospital site as part of routine care based on clinical judgement made by attending physicians such as dengue, *Salmonella*, MTB, HIV, leptospirosis, and bacterial culture. Sera of patients with possible arboviral infection were tested using the dengue NS1 Rapid test (Panbio catalogue#01PF20, Australia) and IgG/IgM Rapid test (PT Biomedika catalogue#AUC-03D07, Indonesia). Patients with possible bacterial infection had samples tested by blood and CSF culture using BacT/ALERT (bioMérieux catalogue#410851, USA) followed by bacterial identification and antimicrobial susceptibility testing using Vitek 2 system (bioMérieux, USA), and for *Salmonella enterica* serotype Typhi (*S*. Typhi) and Leptospira using Uji-Tifoid-IgM^tm^ Rapid Test (PT Pakar Biomedika catalogue#AUC-021D07, Indonesia) and Uji-Leptospira-IgM^tm^ Rapid Test (PT Pakar Biomedika catalogue#AUC-14D09, Indonesia), respectively. Patients who were evaluated for HIV infection were tested with HIV (Ag/Ab) Rapid Test (SD Biosensor, catalogue#10HIV20D, South Korea). Patients with a high clinical suspicion of TB meningitis (TBM) had CSF samples tested by Gram stain, AFB stain and GeneXpert MTB/RIF assay (Cepheid AB, Sweden). For AFB stain assay, sputum smears were stained for acid-fast microscopic examination using the Ziehl-Neelsen stain. Smear-positive specimens were reported semi-quantitatively using the standard scale from the International Union Against Tuberculosis and Lung Disease as recommended by the U.S. CDC [7]. For the GeneXpert MTB/RIF assay, the cartridge was processed according to the manufacturer’s recommendation. Specimens were transported daily to the laboratory at Hasan Sadikin Hospital where they were stored at -80°C. Aliquots of specimens were sent to the Eijkman Institute of Molecular Biology (EIMB) for reference viral and bacteriological assays.

CSF and blood samples underwent molecular analyses at EIMB for a range of pathogens previously associated with AME in Indonesia [8]. Viral RNA and bacterial DNA were extracted using the QIAamp Viral RNA Mini Kit (QIAGEN, Germany) and DNeasy Blood & Tissue Kit (QIAGEN, Germany) respectively in accordance with the manufacturer’s instructions. Extracted RNA was used in chikungunya real-time reverse transcriptase polymerase chain reaction (qRT-PCR) [9] and conventional RT-PCR to detect flaviviruses (Japanese encephalitis virus (JEV), dengue viruses (DENV), Zika virus (ZIKV)) and alphaviruses [10,11]. Positive flavivirus specimens were serotyped using DENV multiplex semi-nested reverse transcription RT-PCR [12]. Amplification of the envelope (E) gene was performed using SuperScript III One-Step RT-PCR System with Platinum *Taq* DNA Pol (Invitrogen-Life Technologies, USA) and serotype-specific primers [13]. In parallel, two-step RT-PCR was conducted to detect the presence of paramyxoviruses (Nipah virus, measles virus, mumps virus) [14], herpesviruses (HSV-1 and 2, CMV, Epstein-Barr virus) [15], enteroviruses (EV) (EV71, Coxsackie virus, influenza A virus [16], and adenoviruses [17]. Positive PCR amplification was followed by sequencing to confirm the result [13]. Sequence reads were assembled using BioEdit and matched to sequences in GenBank database.

CSF and serum were screened by an in-house immunoglobulin M antibody capture enzyme-linked immunosorbent (MAC-ELISA) assay to detect DENV- and JEV-specific antibodies [18,19]. Primary and secondary DENV infections were discriminated using Dengue Virus IgG DxSelect (Focus Diagnostic, USA). IgM antibody for CMV was determined by SERION ELISA classic Cytomegalovirus IgM kit (Institut Virion\Serion GmbH, Germany) in both CSF and sera following the manufacturer’s instructions. HIV status was established for those with positive CMV results. Virus culture by inoculation onto Vero E6 (African green monkey kidney epithelial) cells was performed in those samples positive for arboviruses by PCR. A real-time PCR (qPCR) method, using a specific pair of primer and probes targeting lytA and hpd gene [20], was performed for detection of *S*. *pneumoniae* and Hib respectively, pathogens frequently associated with meningitis. *Salmonella* Typhi PCR assay was conducted targeting flagellin gene following previously published method [21].

Positive cases were classified as confirmed or highly probable CNS infection based on laboratory criteria. A pathogen was confirmed as the etiological agent if it was present in CSF by culture and/or RT-PCR or detection of IgM antibodies in CSF. A highly probable infection was one where a pathogen was positive by culture and/or PCR in blood only. Probable TBM was defined by a Marais Score of ≥12 (imaging available) or ≥10 (imaging not available) when MTB was not detected in CSF.

## Results

**Fig 1:**
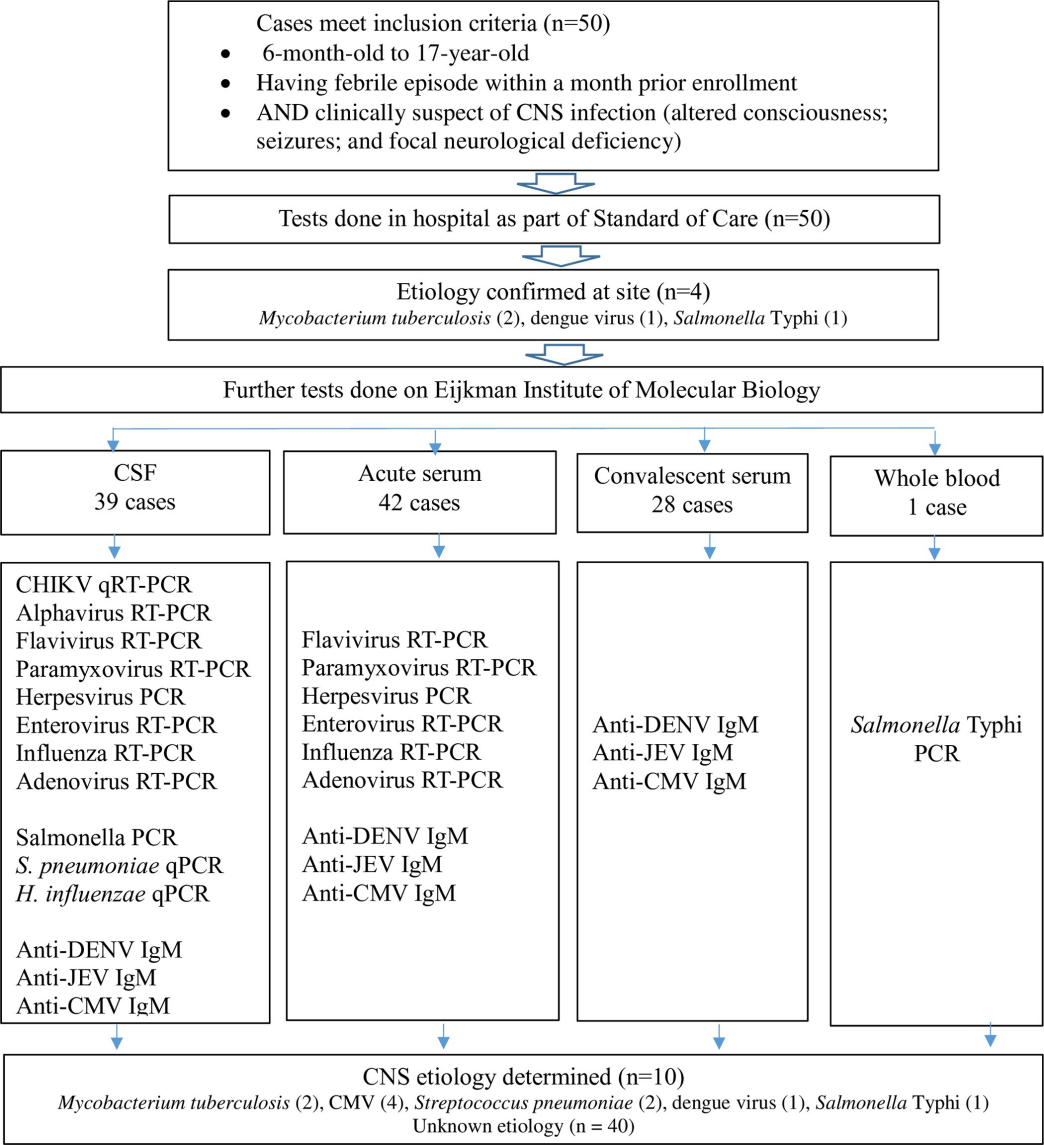
Patient enrollment and testing algorithm of CNS infection study.

This prospective study performed in two hospitals in West Java from October 2017 to November 2018 included 50 hospitalized children meeting the study case definition for suspected CNS infection, 38 from Hasan Sadikin Hospital and 12 from Ujung Berung Hospital. Enrollment of patient and testing panels are illustrated in Fig 1. The demographic and clinical characteristics of the patients included in the study are outlined in Table 1. The median patient age was 5.5 years. There were more male than female children (64.0%). Blood was obtained in 50/50 and CSF in 39/50 children with consenting for routine analysis as per hospital regulations. From 50 cases, some etiological agents were identified on site following standard of care diagnostic assays including 2 MTB cases, 1 dengue case, and 1 *Salmonella* Typhi case. Forty-six cases not suspected of TB and with available CSF and/or blood specimens were forwarded to EIMB for pathogen confirmation or identification. The viral and bacterial diagnostic assays performed and pathogens identified were summarized in Table 2. CNS infection etiology was determined in 10 of 50 (20%) children. The most common pathogen was CMV (4/50), followed by *S*. *pneumoniae* (2/50).

**Table 1:** Clinical and laboratory data of pediatric patients in general and with confirmed etiology of CNS infection, Bandung, Indonesia.

Patient general characteristics	All cases (n = 50)	CMV (n = 4)	DENV (n = 1)	*Salmonella* Typhi (n = 1)	*S*. *pneumoniae* (n = 2)	*M*. tuberculosis** (n = 2)	Unknown (n = 40)
Male	32 (64%)	2	1	1	0	0	28
Female	18 (36%)	2	0	0	2	2	12
Age^a^ (year-old)	5.5 (0.1–15.6)	2.8 (1.9–5.7)	7.32	12.1	3.34 (0.83–5.86)	10.71 (7.07–14.34)	5.30 (0.12–15.61)
Age category							
Infant (< 1 yo)	7 (13.7%)	0	0	0	1 (50%)	0	6 (15.0%)
Toddler (1–2 yo)	12 (23.5%)	2 (50%)	0	0		0	10 (25.0%)
School Age (3–10 yo)	20 (39.2%)	2 (50%)	1	0	1 (50%)	1 (50%)	15 (37.5%)
Adolescent (11–17 yo)	11 (21.6%)	0	0	1 (100%)	0	1 (50%)	9 (22.5%)
Clinical symptoms							
Days of neurological symptom onset before enrollment*	2 (1–31)	1 (1–2)	2	1	1.5 (1–2)	6.5 (3–10)	2 (1–31)
Days of fever before and during hospitalization	8.0 (1–96)	6.5 (1–16)	6	18	6 (2–10)	57 (10–104)	7 (0–96)
N cases (percentage)							
Fever on admission and or history of fever	50 (100.0%)	2 (50%)	1 (100%)	1 (100%)	1 (50%)	1 (50%)	34/49 (69.4%)
Headache	9/49 (18.4%)	4 (100%)	0	0	0	1 (50%)	31/49 (63.3%)
Vomiting	20 (40.0%)	1 (25%)	0	0	0	1 (50%)	17 (42.5%)
Loss of consciousness	41 (82.0%)	4 (100%)	1 (100%)	1 (100%)	2 (100%)	2 (100%)	31 (77.5%)
Seizures	37 (74.0%)	3 (75%)	0	0	1 (50%)	2 (50%)	31 (77.5%)
Paresis	6 (12.0%)	1 (25%)	0	0	0	1 (50%)	4 (10.0%)
Physical Examination							
Glasgow Coma Scale^a^ (n = 47)	12 (8–15)	12 (9–13)	14	13	11 (10–12)	10.5 (9–12)	12 (8–15)
Mild (GCS 13–15)	22/47 (46.8%)	2 (50%)	1 (100%)	1 (100%)	0	0	18 (45.0%)
Moderate (GCS 9–12)	24/47 (51.1%)	2 (50%)	0	0	2	2	18 (45.0%)
Severe (GCS 3–8)	1/47 (2.1%)	0	0	0	0	0	1 (2.5%)
Cranial nerve palsy	10/48 (20.8%)	1 (25%)	0	1 (100%)	0	1 (50%)	7 (17.5%)
Neck stiffness	22/46 (47.8%)	2 (50%)	0	1 (100%)	2 (100%)	2 (100%)	15/36 (41.7%)
Motor deficit	15/49 (30.6%)	1 (25%)	0	1 (100%)	0	1 (50%)	12/49 (24.5%)
**CSF profile (n = 47)**							
Leucocytes^a^ (cells/μL) (n = 46)	3 (0–1,319)	6 (2–170)	4	2	661 (3–1,319)	99.5 (39–160)	3 (0–380)
Polymorphonuclear^a^ (%) (n = 46)	7.5 (0–100)	31.5 (0–50)	0	0	26 (0–52)	11 (8–14)	5.5 (0–100)
Protein^a^ (mg/dL) (n = 43)	32 (18.6–800.0)	30.4 (20.0–800.0)	26.9	26.4	265.1 (19.6–510.6)	130.2 (74.0–186.5)	32.0 (18.6–420.0)
CSF glucose (mg/dL)	70 (3.0–169.0)	82.5 (4.0–99.0)	98.0	58.0	39.0 (3.0–75.0)	37.3 (34.6–40.0)	70.0 (8.0–169.0)
CSF and blood glucose ratio^a^ (n = 20)	0.67 (0.02–1.52)	0.40 (0.3–0.76)	0.7	0.69	0.32 (0.03–0.61)	0.37 (0.36–0.37)	0.71 (0.02–1.52)
**Peripheral blood Examination (n = 50)**							
Hemoglobin (g/dL)	11.5 (6.3–15.7)	10.45 (10.0–15.5)	12.2	12.2	10.85 (8.7–13.0)	11.85 (11.5–12.2)	11.5 (6.3–15.7)
Leucocytes (cells/μL)	9,040 (1,640–37,030)	13,560 (5,060–15,100)	5,570	9,000	10,825 (6,680–14,970)	9,855 (9,040–10,670)	9,055 (1,640–37,030)
Thrombocytes (x10^3^cells/μL)	277.5 (15–674)	212.5 (65–228)	196	74	320.5 (268–373)	416 (250–582)	295 (15–674)
Neutrophils (%)	65.5 (1.0–91.0)	5 (1–75)	74	75	70.5 (54–87)	74 (69–79)	65 (28–91)
Lymphocyte (%)	24.0 (2.0–91.0)	68 (19–91)	18	20	25.5 (10–41)	13 (9–17)	25 (2–66)
Neutrophil lymphocyte count ratio (NLCR)	2.83 (0.07–15.18)	2.01 (0.07–3.95)	4.11	3.75	5.01 (1.32–8.70)	6.42 (4.06–8.78)	2.6 (0.42–15.18)
Absolute lymphocyte count (ALC) (cells/μL)	2,183 (229–8,987)	3,008 (2,576–3,440)	1,002	1,800	2,117.5 (1,498–2,738)	1,248 (960–1,536)	2,234.5 (229–8,987)

Notes: Data presented in number (percentage).

^a^Data presented in median, range.

*Duration from first occurrence of neurological symptoms to admission to hospital. Three patients were admitted due to non-neurologic disorders; however, they started experiencing neurologic symptoms during hospitalization.

**1 case of TBM confirmed with GeneXpert MTB/RIF and another possible case with Marais Score.

**Table 2:** Result of pathogen specific laboratory assays of CNS infection study in children under age of 18 years in Bandung, Indonesia 2017–2018.

Specific pathogen test	Number of positive case/total cases tested	CMV (n = 4)	DENV (n = 1)	*Salmonella* Typhi (n = 1)	*S*. *pneumoniae* (n = 2)	*M*. tuberculosis** (n = 2)	Unknown (n = 40)
CSF bacterial culture	0/33	0/4	0/1	0/1	0/2	0/2	0/23
Gram stain on CSF	0/36	0/3	0/1	0/1	0/1	0/1	0/29
AFB stain on CSF	0/32	0/2	0/1	0/1	0/1	0/0	0/27
Bacterial culture on blood	1/22	0/2	0/1	1/1	0/1	0/0	0/17
Dengue NS1 RDT	0/16	0/2	0/0	0/0	0/0	0/0	0/14
Dengue IgM RDT	2/23	0/1	1/1	0/1	0/2	0/1	1/17
Dengue IgG RDT	6/23	0/1	0/1	0/1	1/2	0/1	5/17
*Salmonella* Typhi IgM RDT	1/22	0/1	0/1	0/1	0/2	0/1	1/16
Leptospira IgM RDT	0/21	0/1	0/1	0/1	0/2	0/1	0/15
HIV (Ag/Ab) RDT	0/10	0/4	0/0	0/0	0/0	0/0	0/6
**Virus Molecular**							
Flavivirus RT-PCR	1/40	neg	1 pos (in CSF, acute serum)	nd	neg	nd	neg
Herpesvirus PCR	3/39	3 pos (CSF)	neg	nd	neg	nd	neg
**Virus Serology**							
Anti-DENV IgM	0/39	neg	1 pos (convalescence serum)	nd	neg	nd	neg
Anti-JEV IgM	0/39	neg	neg	nd	neg	nd	neg
Anti-CMV IgM	1/40	1 pos (serum)	neg	nd	neg	nd	neg
**Bacterial Molecular**							
Salmonella PCR	0/1	neg	nd	neg	neg	nd	neg
*H*. *influenzae* qPCR	0/35	neg	nd	nd	neg	nd	neg
*S*. *pneumoniae* qPCR	2/35	neg	nd	nd	2 pos (CSF)	nd	neg

**Note:** Pos—positive; neg—negative; nd—not done

**1 case of TBM confirmed with GeneXpert MTB/RIF and another possible case with Marais Score

CMV was confirmed using PCR in CSF sample in 3 of 4 cases, all with negative IgM results. One patient with confirmed CMV infection by PCR in CSF was preliminarily diagnosed as viral encephalitis and treated appropriately with acyclovir; this patient recovered completely. Another person initially diagnosed with TB meningitis also had a complete recovery. The third individual, presenting with cerebral infarction confirmed by computerized tomography (CT) scan, had neurological sequelae (paresis) at 3-month follow-up. The forth case with IgM in serum was diagnosed only as a suspected case as IgM may last for months after primary CMV infection; CSF IgM was borderline and the case was fatal. All CMV positive patients, including the fatal case, were immunocompetent (HIV negative) children with ages ranging from 1.9 to 5.7 years.

*Salmonella* Typhi, was found in a 12 yr-old boy confirmed by positive blood culture using BacT/ALERT and serotype was identified using Vitek 2 system. The CSF was negative for *S*. Typhi by PCR assay. The CT scan of this patient revealed suggestive meningitis with multiple tuberculoma. The patient was presumptively diagnosed as typhoid encephalopathy with CSF cell count of 2 cells/μL mononuclear cells, glucose 58 mg/dL, protein 26.4 mg/dL, negative Pandy and GeneXpert MTB/RIF assays. Blood glucose was 91 mg/dL with CSF/blood glucose ratio of 0.64. He was treated with intravenous ceftriaxone for 8 days. The patient was found to be doing well at 3 months follow up with some visual disability and without any other neurological deficit.

*S*. *pneumoniae* was confirmed in two cases by qPCR. The first patient, a 1 yr-old girl, had CSF findings typical of bacterial meningitis including polymorphonuclear pleocytosis, low glucose level and high protein content. However, CSF Gram stain was negative. She was treated with empiric antibiotic treatment therapy (ceftriaxone) and recovered completely. The second case, a 5 yr-old girl, was initially diagnosed as viral encephalitis based on normal CSF findings (leucocyte count 3 cells/μL; protein CSF 19.6 mg/dL; glucose CSF 75 mg/dL). She was treated with acyclovir as well as ceftriaxone and was discharged 4 days after admission.

Dengue was confirmed by PCR and IgM ELISA based on both CSF and serum samples in one individual with encephalitis admitted with fever, decreased consciousness, and coffee ground vomiting. The patient was provided with supportive treatment including adequate electrolyte infusion and antipyretic drugs and recovered completely without any disability [22].

Tuberculous meningitis was clinically suspected in sixteen cases but only one case was confirmed by GeneXpert MTB/RIF in CSF. All suspect patients were treated with a first line anti-TB regimen containing rifampicin, isoniazid, ethambutol and pyrazinamide. The confirmed TBM case was reported to have recovered completely on 3-month follow-up. Another patient diagnosed as probable TBM based on Marais Score, was also treated accordingly with first line TB treatment. Additionally, *Klebsiella pneumoniae* was isolated through blood culture from this patient while CSF culture was sterile. She was treated with ceftriaxone and then replaced with amikacin after antibiotic sensitivity test result was obtained. The patient expired after 79 days of hospitalization despite high level ICU care.

Overall case fatality was 20% (10/50), while 15 (30%) were discharged with neurological sequelae including one patient with intellectual disability (Table 3). No evidence of co-infection was found in any of the CSF samples. All patients had a history of receiving Hib-pentavalent vaccine but not 13-valent pneumococcal conjugate vaccine (PCV 13) nor the JEV vaccine.

**Table 3:** Disease outcome of CNS infection study in children under age of 18 years in Bandung, Indonesia 2017–2018.

Patient characteristics	CMV	DENV	*Salmonella* Typhi	*S*. *pneumoniae*	*M*. tuberculosis**	Unknown
	(n = 4)	(n = 1)	(n = 1)	(n = 2)	(n = 2)	(n = 40)
Duration of hospitalization (days)	10.5 (4–47)	7	9	7.5 (4–11)	46.5 (14–79)	8.5 (2–25)
Treatment						
Antiviral treatment	0	0	0	1 (50%)	0	2/38 (5.3%)
Antibiotic treatment	3/3 (100%)	1 (100%)	1 (100%)	2 (100%)	2 (100%)	28/38 (73.7%)
	Ceftriaxone	Ceftriaxone	Ceftriaxone	Ceftriaxone	Ceftriaxone	Ceftriaxone (26/38 (65%))
Anti-tuberculosis	1/3 (33.3%)	0	1 (100%)	0	2 (100%)	18/38 (47.4%)
Nosocomial infection	0/3	0	0	0	0	4/38 (10.5%)
Others	0	0	1 (100%)	1 (50%)	1 (50%)	18/38 (47.4%)
**Outcome at hospital discharge**						
Complete recovery	0	0	0	1	1	7
Recovered with sequelae	2	1	1	1	0	18
Neurological sequelae***	2	1	1	1		N/A
Intelectual disability						N/A
Condition unchanged	0	0	0	0	1	1
Data not available	1	0	0	0	0	10
Death	1	0	0	0	0	4
**Outcome at 3-month follow-up**						
Complete recovery	2	1	0	1	1	22
Recovered with sequelae	1	0	1	1	0	9
Neurological sequelae***	1	0	1	1	0	8
Intelectual disability	0	0	0	0	0	1
Condition unchanged	0					1
Withdraw	0					1
Lost to follow up	0					1
Death	1	0	0	0	1	6

Data presented in number of positive results/number of cases analyzed (percentage).

**One case of MTB confirmed with GeneXpert MTB/RIF and another possible case with Marais Score

***Neurological sequelae is including headache, paresis, seizure, decrease of consciousness, visual problem, hearing disability, and verbal disability; N/A: data not available

## Discussion

The present study provides the first comprehensive data describing the etiological agents of AME and outcome of hospitalized children from West Java, Indonesia. In our study, CMV was the most frequently detected pathogen, which was diagnosed by the presence of CMV DNA (n = 3) or IgM antibodies (n = 1), the latter a suspected case without CSF confirmation. CNS infection with CMV is more prevalent in developed countries and is commonly associated with HIV [23], as CMV encephalitis in the immunocompetent host is rarely reported [24]. The presence of CMV DNA is most likely to be confirmatory as there is less tendency of the virus to remain latent within lymphocytes [25]. Although CMV encephalitis is typically reported in infants [26], the children affected in our study were older than 2 years. Prominent features associated with CMV encephalitis such as altered consciousness and seizures, were seen in 100% (4 of 4) and 75% (3 of 4) of positive individual, respectively. Although it is possible that the presence of CMV could be coincidental detection due to re-activation of latent infection [27], it was most likely the causative agent for the four immunocompetent patients as reported in an earlier study [28]. One of our study case subjects with recent CMV infection who had IgM in serum was fatal. The corresponding CSF was borderline for IgM and extensive etiological investigations could not be undertaken to confirm the diagnosis.

In Indonesia, *S*. *pneumoniae* remains a common bacterial cause of AME as identified in our study; PCV 13 immunization was not reported in any of the enrolled children as its use is not widespread in Indonesia. Two cases of *S*. *pneumoniae* was confirmed by PCR, similar to earlier reports from Indonesia [29], Vietnam [2], Thailand [17], Bangladesh [30] and Cambodia [31]. Although both received empirical antibiotic treatment, only one case showed typical features of bacterial meningitis in CSF analysis. Both were negative for direct microscopy in CSF which could be attributed to prior antibiotic therapy before admission.

*Salmonella* Typhi meningitis seen in developing countries is uncommon in children above the age of two [32]. Our case of probable *S*. Typhi in an adolescent person would have been missed if blood culture was not performed suggesting this procedure should be considered in children with neurological disease with no other identified etiology. The common clinical manifestations of *S*. Typhi meningitis such as seizures, vomiting and diarrhea [33] were not reported in this case. Salmonella culture, the gold standard for diagnosis, was positive in blood but not conducted in CSF as per hospital routine. CSF PCR performed retrospectively was negative and CSF findings were non-specific, similar to an earlier report [34] although PCR positivity was reported to persist after 3 weeks [35]. Although *Salmonella* Typhi meningitis could be associated with severe complications including hydrocephalus [32], our study individual was discharged 9 days after admission and reported to have visual deficit on 3-month follow-up. Despite its high morbidity and mortality in endemic areas, *S*. Typhi meningitis could be considered an infection with favorable prognosis due to its success with early initiation of third generation cephalosporin.

Dengue encephalitis is reported with increasing frequency in endemic areas [36]. One individual in our study with gastrointestinal bleeding followed by a full recovery was confirmed as having DENV-1 serotype by PCR [22], highlighting that DENV should be considered as an etiologic agent in endemic areas especially in those with suggestive clinical and laboratory features including rash, petechiae, hemorrhage, thrombocytopenia and leucopenia.

In Indonesia, pediatric TB accounts for 9% of total TB cases [37] with pediatric TBM seen more commonly than in adults. In our study, the individual with the single confirmed case of TBM recovered without sequelae at 3-month follow-up, most likely due to early initiation of empirical anti-TB therapy. Another case diagnosed with probable TB meningitis based on Marais Score, expired after a prolonged hospitalization in spite of empirical anti-TB therapy.

*Haemophilus influenza b*, a common cause of bacterial meningitis, was not seen at all in our study most likely due to low incidence as seen in other studies [38] and the introduction of *Hib* vaccine into the National Immunization Program in 2013.

There were 8 fatalities in our study, 5 of which were during hospitalization including one with CMV. One of the patients with TB was discharged against medical advice with unchanged condition and died one month after she went home. Information on the underlying cause of death for other fatal cases was not available. The results from our study were quite different from the regional studies where enteroviruses (EV) and vector-borne pathogens like JEV and DENV were reported frequently [39–42]. JEV has been reported as the leading cause of acute encephalitis in children in Southeast Asia [43,44]; however, JE burden in West Java, Indonesia was not confirmed here. Although JEV vaccine is not routinely practiced in West Java, one of the provinces with historically high incidence of JE [45], JEV encephalitis was not seen in any of the study case subjects. Although EV are increasingly recognized as important pathogens for pediatric CNS infections, none was identified in the present study probably due to the absence of supportive respiratory and stool specimens [46]. In addition, influenza and adenoviruses, common pediatric CNS etiologic agents [23] were not identified in our study most likely due to lack of respiratory specimens. The small sample size as well as the seasonality of these viruses may have been factors limiting the presence of these agents. Toxoplasma and Cryptococcus, major opportunistic infections in HIV/AIDS, were not tested as the patients were screened by HIV rapid test and determined to be negative.

In our study, a pathogen was established in only 20% of enrolled children, highlighting the challenges of obtaining accurate diagnosis of CNS infection in Indonesia. The difficulties lie in the lack of capacity in conducting proper investigation and management of CNS infection. Lumbar puncture is not conducted as often as necessary in many settings due to lack of awareness of the early symptoms of CNS infection as well as competent personnel. PCR for TB (GeneXpert MTB/RIF) recently introduced for TB meningitis, is still unfamiliar to many pediatricians. Although a more comprehensive pathogen identification in CSF can be seen in many developing countries [47], viral diagnostic testing for CMV, enterovirus, herpesvirus remains largely unavailable in many hospitals.

There were some limitations to the study including equipment, temporal, and geographical restrictions. The study was conducted in two sites in West Java over a short duration with a sample size too small for an accurate statistical analysis. This would prevent the extrapolation of the data to the entire country. TBM with atypical features might have been missed as not all cases were tested by molecular assays and serological testing was focused only on arboviruses which may have led to an underestimation of other viral and bacterial causes of AME. In addition the inclusion of diagnostics for pediatric autoimmune-mediated encephalitis [41], and collection of supportive specimens such as respiratory and rectal swabs could have contributed to a wider identification of causal pathogens. Finally, neuroimaging was not performed that could have assisted in the diagnosis; a common limitation in developing countries due to limited resources.

## Conclusion

Although CNS infections are among the most devastating infectious diseases and a major cause of pediatric death and disability worldwide, the pathogens responsible have not been well studied in Indonesian children. Our study on pediatric CNS infections in West Java revealed several CMV positive cases in immunocompetent children diagnosed by the presence of CMV DNA. Although pathogens such as CMV, DENV, *S*. *pneumoniae* and *Salmonella* Typhi were found to be associated with AME in our study, no reliable differentiation could be found between viral and bacterial etiology based on clinical and lab manifestations. Despite the small sample size and low percentage of pathogens identified, the data from the present study may be applicable in increase awareness and better understanding among physicians to develop preventive strategies and therapeutic consideration for CNS infections in the Indonesian pediatric population and for researchers to identify new and more effective diagnostic methods of pathogen detection.

## Supporting information

S1 File Assessment Tool at Discharge and Follow up at 3 Months After Enrollment. (DOCX)
