# Supplementary material for: Central nervous system infection in a pediatric population in West Java
Source: PLoS Negl Trop Dis. 2023 Nov 27;17(11):e0011769. doi: 10.1371/journal.pntd.0011769 (PMC10703213; doi:10.1371/journal.pntd.0011769)
Supplement: S1 File — (DOCX) [file pntd.0011769.s001.docx]

# **S1 File. Assessment Tool at Discharge and Follow up at 3 Months After Enrollment**

# **A. CONDITION AT DISCHARGE FROM HOSPITAL**

1. Study ID : _______________________
2. Researcher name : _______________________
3. Visit date : __ __ / __ __ __ /__ __ __ __ (dd/mmm/yyyy)

# Socio demographic

| Demographic data | | |
| --- | --- | --- |
|  | Date of hospital discharge | _ _ /_ _ _ /_ _ _ _ (dd/mmm/yyyy) |
|  | Length of hospitalization | _ _ /_ _ _ /_ _ _ _ (dd/mmm/yyyy) |

# Medical History

| Fever Description | | | | |
| --- | --- | --- | --- | --- |
|  | End date of fever | : _ _ /_ _ _ /_ _ _ _ (dd/mmm/yyyy) | | 🞏 Ongoing |
|  | Type of fever in hospital | 🞏 Continous | 🞏 Remittent | 🞏 Intermittent |

# Clinical Diagnosis : _______________________________________

# Differential Diagnosis: _______________________________________

# Clinical outcome:

| 🞏 Recovered | 🞏 Recovered with sequelae | 🞏 Unchanged | 🞏 Worsened | 🞏 Death |
| --- | --- | --- | --- | --- |

# Presence of sequelae

| Is there any presence of sequelae? | | | | | |
| --- | --- | --- | --- | --- | --- |
|  | Sequelae | Yes | No | Unknown |  |
|  | Hearing disability | 🞏 | 🞏 | 🞏 |  |
|  | Visual disability | 🞏 | 🞏 | 🞏 |  |
|  | Verbal disability | 🞏 | 🞏 | 🞏 |  |
|  | Paresis | 🞏 | 🞏 | 🞏 |  |
|  | Intellectual disability | 🞏 | 🞏 | 🞏 |  |
|  | Recurrent seizures | 🞏 | 🞏 | 🞏 |  |
|  | Others: | 🞏 | 🞏 | 🞏 |  |

# **B. FOLLOW UP AND FINAL OUTCOME AT 3 MONTHS AFTER ENROLLMENT**

1. Study ID : _________________
2. Researcher name : _____________________
3. Visit date : _ _ /_ _ _ /_ _ _ _ (dd/mmm/yyyy)

| Fever Description | | | |
| --- | --- | --- | --- |
|  | End date of fever | _ _ /_ _ _ /_ _ _ _ (dd/mmm/yyyy) | 🞏 Ongoing |

| Neurological Symptoms | | |
| --- | --- | --- |
| 1. End date of headache | _ _ /_ _ _ /_ _ _ _ (dd/mmm/yyyy) | 🞏 Ongoing |
| 1. End date of seizures | _ _ /_ _ _ /_ _ _ _ (dd/mmm/yyyy) | 🞏 Ongoing |
| 1. End date of decrease of consciousness | _ _ /_ _ _ /_ _ _ _ (dd/mmm/yyyy) | 🞏 Ongoing |
| 1. End date of irritability | _ _ /_ _ _ /_ _ _ _ (dd/mmm/yyyy) | 🞏 Ongoing |
| 1. End date of paresis | _ _ /_ _ _ /_ _ _ _ (dd/mmm/yyyy) | 🞏 Ongoing |

# Presence of sequelae

| Is there any presence of sequelae? | | | | |
| --- | --- | --- | --- | --- |
|  | Sequelae | Yes | No | Unknown |
|  | Hearing disability | 🞏 | 🞏 | 🞏 |
|  | Visual disability | 🞏 | 🞏 | 🞏 |
|  | Verbal disability | 🞏 | 🞏 | 🞏 |
|  | Paresis | 🞏 | 🞏 | 🞏 |
|  | Intellectual disability | 🞏 | 🞏 | 🞏 |
|  | Recurrent seizures | 🞏 | 🞏 | 🞏 |
|  | Others: | 🞏 | 🞏 | 🞏 |

| Final Status | | | | | |
| --- | --- | --- | --- | --- | --- |
| 17. | End of study status | 🞏 Patient completed the study | | Final contact date: _ _ /_ _ _ /_ _ _ _ (dd/mmm/yyyy) | |
|  |  | 🞏 Patient died | | Date of death : _ _ /_ _ _ /_ _ _ _ (dd/mmm/yyyy) | |
|  |  |  |  | Cause of death: | |
|  |  |  |  | 🞏 Neurological | |
|  |  |  |  | 🞏 Non-neurological | |
|  |  | 🞏 Patient did not complete  study | | Date of last contact: _ _ /_ _ _ /_ _ _ _ (dd/mmm/yyyy) | |
|  |  |  |  | Reason for not completing study: | |
|  |  |  |  | 🞏 Lost to follow-up | |
|  |  |  |  | 🞏 Withdrawn by physician | |
|  |  |  |  | 🞏 Family withdraw consent | |
|  |  |  |  | 🞏 Other: __________ | |
| 18. | Outcome | 🞏 recovered | 🞏 recovered with sequelae | 🞏 Condition unchanged | 🞏 death |
